# MicroRNA-15a/16-1 cluster located at chromosome 13q14 is down-regulated but displays different expression pattern and prognostic significance in multiple myeloma

**DOI:** 10.18632/oncotarget.5681

**Published:** 2015-10-12

**Authors:** Fei Li, Yan Xu, Shuhui Deng, Zengjun Li, Dehui Zou, Shuhua Yi, Weiwei Sui, Mu Hao, Lugui Qiu

**Affiliations:** ^1^ State Key Laboratory of Experimental Hematology, Institute of Hematology and Blood Disease Hospital, Chinese Academy of Medical Sciences and Peking Union Medical College, Tianjin 300020, China; ^2^ Department of Hematology, The First Affiliated Hospital of Nanchang University, NanChang 330006, China

**Keywords:** multiple myeloma, miR-15a, miR-16-1, prognosis

## Abstract

MiRNA-15a/16-1 cluster located at chromosome 13q14 has been confirmed to regulate critical genes associated with cell proliferation, apoptosis and drug resistance in multiple myeloma (MM). However, little is known about their expression pattern and prognostic value in MM patients. In this study, we have analyzed the expression levels of miR-15a/16-1 in 117 MM patients (90 newly diagnosed, 11 relapsed and 16 remission patients) and 19 health donors (HDs) by quantitative real-time PCR. Our results indicated that the expression levels of miR-15a and 16-1 were down-regulated in newly diagnosed MM patients as compared to HDs (*P* = 0.025; *P* < 0.001) and independent of del(13q14). Downregulation of miR-15a was significantly associated with disease progression and poor prognosis while miR-16-1 seemed to be a good diagnostic marker to distinguish MM from HDs with area under the curve (AUC) of 0.864, sensitivity of 100% and specificity of 73%. Furthermore, patients with miR-15a < 2.35 (low expression group) had significantly shorter PFS (*P* < 0.001) and OS (*P* < 0.001). After adjustment of the established prognostic variables including del(13q), del(17p), amp(1q21) and high risk genetic abnormality, low miR-15a expression (<2.35) was still a powerful independent predictor for PFS (*P* = 0.008) and OS (*P* = 0.038). In addition, miR-15a combined with high β2-MG and high risk genetic abnormality can further identify the high-risk subpopulations. Therefore, our data suggest that the expression patterns of miR-15a/16-1 are different in MM patients, and miR-15a seems to be linked with disease progression and prognosis while miR-16-1 acts as a valuable diagnostic marker.

## INTRODUCTION

Multiple myeloma (MM) is a clonal plasma cell malignancy characterized by complex chromosomal instability. It involves both numerical and structural aberrations that are recognized as the most important factors for providing the potential prognostic relevance and guidance for the therapeutic strategies [1, 2]. The routine evaluation factors consist of 17p deletion (del(17p)), t(4;14) and t(14;16) detected by fluorescence *in situ* hybridization (FISH) [2]. In recent years, increasing evidences have shown that miRNAs play a critical role in the pathogenesis of human tumors and are also found to be valuable markers for predicting the diagnosis, risk-stratification and clinical outcomes [3, 4]. In 2013, Wu *et al*. first suggested that miRNAs could be built into molecular diagnostic strategies for improving the International Staging System (ISS)/FISH-based risk stratification and were even independent of gene expression profiles (GEP) signatures based approach that has been considered as important predictors to classify the different clinical outcomes in MM patients [5]. To date, although many miRNAs dysregulations in MM have already been reported, their expression patterns and prognostic impacts on MM patients remain elusive.

MicroRNAs (miRNAs) are small, approximately 18–22-nucleotide non-coding RNA molecules that negatively regulate posttranscriptional gene expression by binding to the 3′-untranslated region of their target transcripts. MiR-15a/16-1 cluster, closely located at chromosome 13q14, was considered to have similar tumor suppressor functions involved in cell differentiation, proliferation, apoptosis or angiogenesis in several human tumors including MM [6–10]. Our previous studies indicated that miR-15a/16-1 downregulation contributed to the myeloma pathogenesis and mediates drug resistance in myeloma cells [11, 12]. However, their expression patterns and the ability to predict diagnosis and clinical outcome in MM patients are still unclear. Therefore, in this study we investigated the expression of miR-15a/16-1 and their subsequent clinical predictive values in MM patients.

## RESULTS

### Patient's characteristics

A total of 90 newly diagnosed MM patients including 44 IgG type, 26 IgA type, 6 IgD type and 14 light chain type were enrolled in this study. Among these patients, 55 were males and 35 were females. The median age of the patients was 58 years old (range, 40–79 yr). The clinical characteristics and miR-15a/16-1 expression levels of the 90 newly diagnosed patients are shown in Table 1. Among the newly diagnosed patients, 49 received thalidomide-based treatment (Arm A) and 41 received bortezomib-based treatment (Arm B). There were no significant differences in clinical and cytogenetic characteristics between Arm A and B (Supplemental data, Table 1).

**Table 1:** Clinical characteristics and miRNA-15a/-16-1 expression in newly diagnosed patients

Characteristics	N (%)	miRNA-15a (mean ± SD)	*P-value*	miRNA-16-1 (mean ± SD)	*P-value*
Durie-Salmon stage			0.325		0.196
I-II	10 (11.6)	3.05 ± 1.47		2.81 ± 0.75	
III	76 (88.4)	3.44 ± 0.93		2.85 ± 0.73	
ISS stage			0.532		0.240
I-II	37 (43.0)	3.42 ± 1.09		2.80 ± 0.76	
III	49 (57.0)	3.38 ± 0.92		2.99 ± 0.76	
LDH, U/L			0.668		0.661
≥ 220	18 (22.5)	3.42 ± 1.08		2.91 ± 0.87	
< 220	62 (77.5)	3.38 ± 0.99		2.83 ± 0.75	
Renal lesion			0.577		0.179
no	66 (75.9)	3.40 ± 0.99		2.70 ± 0.33	
yes	21 (24.1)	3.35 ± 1.00		3.02 ± 0.86	
Del(13q)			0.436		0.337
no	60 (66.7)	3.40 ± 1.03		2.81 ± 0.67	
yes	30 (33.3)	3.43 ± 0.93		2.84 ± 0.87	
Del(17p)			0.874		0.663
no	78 (88.6)	3.50 ± 1.02		2.85 ± 0.76	
yes	10 (11.4)	3.00 ± 0.63		2.58 ± 0.63	
Amp(1q21)			0.091		0.215
no	38 (46.3)	3.59 ± 0.86		2.99 ± 0.80	
yes	44 (53.7)	3.20 ± 1.05		2.70 ± 0.67	
IGH translocation			0.702		0.990
no	34 (39.5)	3.50 ± 1.16		2.80 ± 0.90	
yes	52 (60.5)	3.38 ± 0.89		2.89 ± 0.66	
t(11;14)			0.221		0.860
no	66 (82.5)	3.38 ± 1.04		2.72 ± 0.78	
yes	14 (17.5)	3.42 ± 0.61		2.96 ± 0.47	
t(4;14)			0.474		0.151
no	64 (80.0)	3.41 ± 0.96		2.85 ± 0.78	
yes	16 (20.0)	3.11 ± 0.98		2.70 ± 0.58	
t(14;16)			0.216		0.796
no	74 (93.7)	3.42 ± 0.98		2.87 ± 0.72	
yes	5 (6.3)	2.68 ± 0.66		1.90 ± 0.30	
High-risk [(any t(4;14), t(14;16) or del(17p)]			0.137		0.060
no	60 (73.2)	3.62 ± 0.93		2.85 ± 0.77	
yes	22 (26.8)	3.00 ± 0.88		2.70 ± 0.59	
Disease progression			0.040		0.347
no	60 (69.0)	3.46 ± 0.92		2.97 ± 0.64	
yes	27 (31.0)	3.28 ± 1.13		2.68 ± 0.93	
Dead			0.012		0.145
no	67 (77.9)	3.46 ± 0.91		2.85 ± 0.72	
yes	19 (22.1)	3.06 ± 0.94		2.60 ± 0.62	

ISS, International Staging System

In addition, we also enrolled 11 relapsed and 16 remission (≥VGPR, very good partial remission) MM patients for comparing the miR-15a/16-1 expression at different stages of disease. Due to limited samples and follow-up time, only three paired samples (newly diagnosed and relapsed patients) and a paired of newly diagnosed and remission sample were included in this study. The clinical characteristics of 16 remission patients are described in the Supplementary Table 2.

### MiR-15a and miR-16-1 expression levels are down-regulated and positively correlated in newly diagnosed MM patients

**Figure 1:**
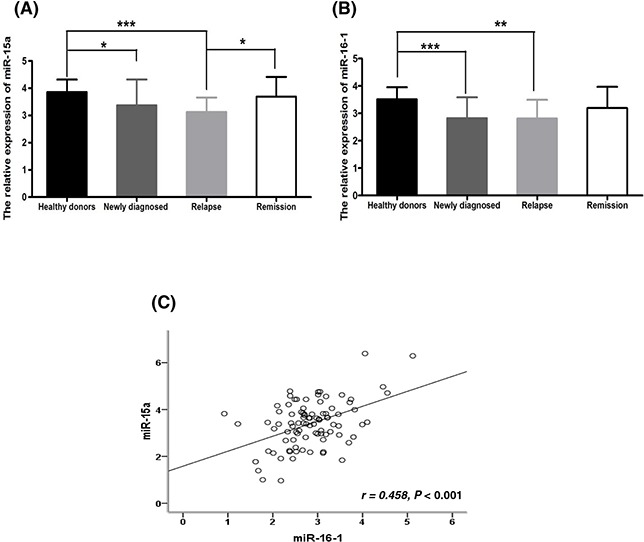
Comparison of miR-15a and miR-16-1 expression among 90 newly diagnosed, 11 relapsed, 16 remission MM patients and 19 healthy donors (HDs) **A.** MiR-15a is obviously down-regulated in newly diagnosed and relapsed patients compared to HDs (*P* = 0.025, *P* < 0.001); however, it restores to HDs level from relapsed to remission status (*P* = 0.017). **B.** MiR-16-1 is obviously down-regulated in newly diagnosed and relapsed patients compared to HDs (*P* < 0.001, *P* = 0.002); however, it didn't restore to HDs levels from relapsed to remission status (*P* = 0.285). **C.** The expression levels of miR-15a and miR-16-1 were positively correlated (r = 0.458, *P* < 0.001). (* means *P* < 0.05; ** means *P* < 0.01; *** means *P* < 0.001).

As shown in Figure 1A, miR-15a expression was significantly down-regulated in newly diagnosed MM patients compared to HDs (3.42 *vs*. 3.75, *P* = 0.025). Similarly, the expression of miR-16-1 also displayed a down-regulated trend between the newly diagnosed patients and HDs (2.83 *vs*. 3.51, *P* < 0.001) (Figure 1B). Since miR-15a and miR-16-1 are closely located on the chromosome 13q14 region, we next analyzed their correlation and observed that the expression levels of miR-15a and miR-16-1 were positively correlated (*r* = 0.458, *P* < 0.001) as seen in Figure 1C.

### Downregulation of miR-15a is linked with disease progression while miR-16-1 seems to be a good diagnostic marker in MM

**Figure 2:**
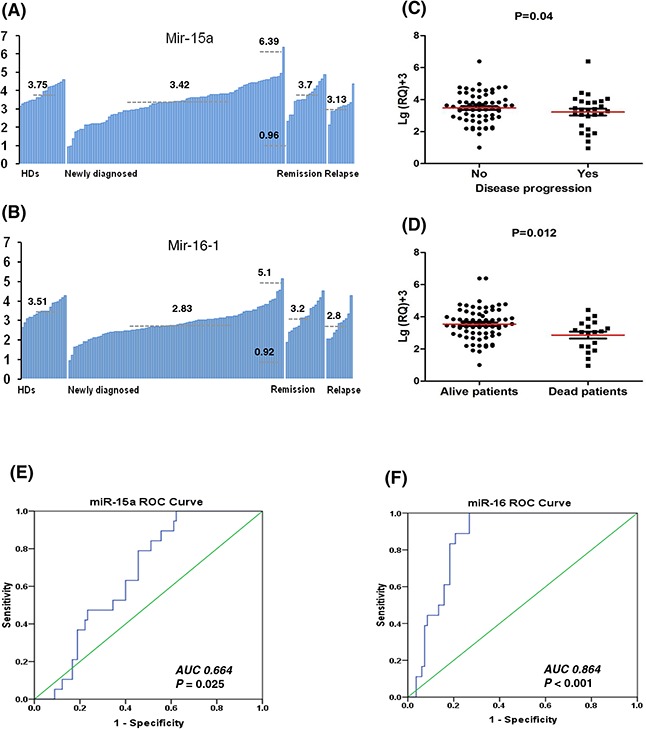
The low miR-15a expression is a good marker for predicting disease progression while miR-16-1 seems to be a good diagnosed marker for distinguishing MM patients from HDs **A.** The expression levels of miR-15a showed more variation with a mean 3.42 and standard deviation (SD) of 1.0 (ranged from 0.96 to 6.39) in newly diagnosed patients; while HDs, remission and relapsed patients with the means of 3.75, 3.70 and 3.13, respectively. **B.** The expression levels of miR-16-1 showed a mean of 2.83 and SD of 0.75 (ranged from 0.92 to 5.1); while HDs, remission and relapsed patients with the means of 3.51, 3.2 and 2.8, respectively. **C.** Patients with disease progression have lower expression levels of miR-15a than patients without progression (*P* = 0.04). **D.** The died patients have lower expression levels of miR-15a than live patients (*P* = 0.012). **E.** The ROC curve of miR-15a showed area under the curve (AUC) of 0.664 (*P* = 0.025). **F.** The ROC curves of miR-16-1 showed AUC of 0.864 (*P* < 0.001), suggesting that miR-16-1 can be used as a better diagnosed marker to distinguish MM patients from HDs than miR-15a.

It is worthy to note, we observed that there was a greater variation in miR-15a expression than in miR-16-1 expression among different newly diagnosed patients. The expression levels of miR-15a in newly diagnosed patients ranged from 0.96 to 6.39, with the mean value of 3.42 and standard deviation (SD) of 1.0, while it ranged from 3.15 to 4.62 in HDs, with the mean value of 3.75 and SD of 0.46 as seen in Figure 2A. In contrast, the expression levels of miR-16-1 in newly diagnosed patients ranged from 0.92 to 5.1, with the mean value of 2.83 and SD of 0.75, while it ranged from 2.64 to 4.28 in HDs, with the mean of 3.51 and SD of 0.45 (Figure 2B). Moreover, it is important to note here that the observed correlation coefficient between the expression levels of miR-15a and miR-16-1 was moderate (r = 0.458), suggesting that there is the potential possibility of difference in their expression pattern and clinical significance although with the similar down-regulation trend in newly diagnosed patients.

To understand the meaning of miR-15a/16-1 downregulation in newly diagnosed MM patients, we further compared the expression levels of miR-15a/16-1 in patients at different stages of disease. The results showed miR-15a was obviously down-regulated in newly diagnosed patients but restored close to HDs levels at the remission phase. However, the levels of miR-15a returned to diagnostic levels upon relapse (Figure 1A and 2A). However, although miR-16-1 was also obviously down-regulated in newly diagnosed and relapsed patients, the levels of miR-16-1 didn't restore to HDs levels at the remission phase (Figure 1B and 2B). We further analyzed the correlation between their expression levels and clinical characteristics. Here, we found that patients with disease progression (*P* = 0.04) (Figure 2C) and death (*P* = 0.012) (Figure 2D) had lower levels of miR-15a. However, we didn't find any correlation between clinical characteristics and miR-16-1 expression.

Next, we compared the ability of miR-15a and miR-16-1 to distinguish MM patients from HDs using receiver operating characteristic (ROC) analysis. The results showed that the levels of miR-16-1 can be used to distinguish MM patients from HDs with area under the curve (AUC) of 0.864 (*P* < 0.001), sensitivity of 100% and specificity of 73% (Figure 2F). While the AUC for miR-15a was only 0.664 (*P* = 0.025) and the sensitivity and specificity were 78.9% and 56.7%, respectively (Figure 2E). These results confirmed that the variation in miR-15a expression in myeloma might be useful in monitoring disease progression, while miR-16-1 could be used as a good diagnostic marker to distinguish MM patients from HDs.

### MiR-15a and miR-16-1 expression levels are independent of the chromosome 13q14 deletion

To further investigate if miR-15a/16-1 downregulation was associated with the deletion of chromosome 13q14, we assessed the expression levels of miR-15a/16-1 in patients with or without deletion of 13q14. Heterozygous del(13q14) was detected in 33.3% (30/90) of the patients. The mean levels of miR-15a expression in patients with and without del(13q14) were 3.43 ± 0.93 and 3.40 ± 1.03 (*P* = 0.436), and the mean levels of miR-16-1 expression in patients with and without del(13q14) were 2.84 ± 0.87 and 2.81 ± 0.67 (*P* = 0.337). These results suggest that miR-15a and miR-16-1expression levels are independent of the deletion of chromosome 13q14.

### MiR-15a downregulation is linked with poor survival in newly diagnosed MM patients

Since we observed the correlation between low miR-15a expression and disease progression and patient survival, we next compared whether patients with different miR-15a levels have potentially different clinical outcomes. Using ROC curve analysis, we defined 2.35 as a cut-off value for miR-15a expression. Seventeen patients with miR-15a expression less than 2.35 were defined as low miR-15a expression group, while seventy-three patients with miR-15a ≥ 2.35 were defined as high miR-15a expression group. The patients were followed for a median time of 15 months (range, 3.0–55.5 months). Four patients did not complete the follow-up examination due to incorrect contact information.

**Figure 3:**
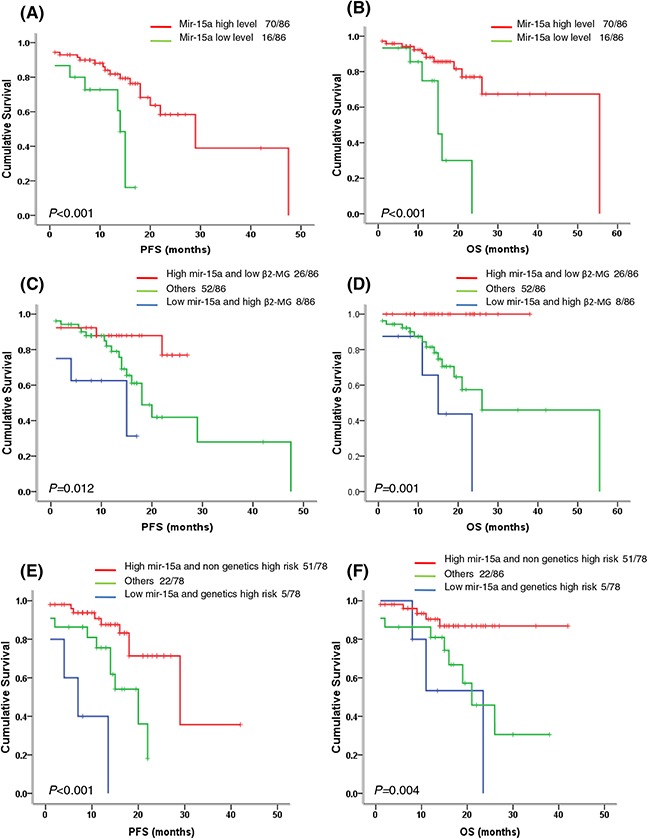
Survival analysis of miR-15a in newly diagnosed MM patients **A–B.** Patients with low miR-15a expression (<2.35) have significantly shortened PFS (*P* < 0.001) and OS (*P* < 0.001). **C–D.** The patients with low miR-15a expression and high β2-MG had the shortest PFS and OS, followed by the patients with low miR-15a expression or high β2-MG, the patients with high miR-15a expression and low β2-MG had relatively better outcomes (*P* = 0.012, *P* = 0.001). **E–F.** The patients with low miR-15a expression and high risk genetics had the shortest PFS and OS, followed by the patients with low miR-15a expression or high risk genetics, the patients with high miR-15a expression and non high risk genetics had relatively better outcomes (*P* < 0.001, *P* = 0.004).

As shown in Table 2 and Figure 3A & 3B, patients with low miR-15a expression (<2.35) had significantly shorter progression free survival (PFS) (14.0 months vs. 29.0 months, *P* < 0.001) and overall survival (OS) (15.0 months vs. 55.0 months, *P* < 0.001) than the patients with high miR-15a expression (≥2.35). Based on the univariate analysis as shown in Table 2, the patients with del(13q), del(17p), amp (1q21), high-risk genetic abnormality also showed shorter survival time than the patients without above abnormalities. Furthermore, the multivariate cox regression model analysis also revealed that miR-15a was an independent prognostic factor for PFS (HR 0.26, 95% CI: 0.09–0.71, *P* = 0.008) and OS (HR 0.28, 95% CI: 0.08–0.93, *P* = 0.038). In addition, the del(17p) and del (13q) also had the independent adverse influence on PFS and OS (Table 3).

**Table 2:** Univariate analysis of risk factors for PFS and OS in 90 newly diagnosed MM patients

Prognostic parameters	Median PFS (months)	*P* value	Median OS (months)	*P* value
Del(13q)		0.019		0.071
Positive	16.0		26.0	
Negtive	29.0		55.5	
Del(17p)		<0.001		<0.001
Positive	14.0		16.0	
Negative	29.0		55.5	
Amp (1q21)		0.013		0.042
Positive	18.0		26.0	
Negative	47.5		55.5	
Cytogenetic abnormality		0.005		0.043
High-risk	20.0		23.5	
Non high-risk	29.0		Not reached	
Mir-15a		<0.001		<0.001
<2.35	14.0		15.0	
≥2.35	29.0		55.0	
Mir-16-1		0.104		0.229
<3.13	20.0		55.0	
≥3.13	Not reached		Not reached	

Cytogenetic high-risk: any t(4;14), t(14;16) or del(17p).

**Table 3:** Multivariate analysis of risk factors for PFS and OS in newly diagnosed MM patients

Prognostic parameter	HR for PFS (95% CI)	*P* value	HR for OS (95% CI)	*P* value
Del(13q)	2.75 (1.06–7.17)	0.039	4.94 (1.40–17.39)	0.013
Del(17p)	4.33 (1.51–12.40)	0.006	4.83 (1.33–17.57)	0.017
Amp (1q21)	2.43 (0.93–6.39)	0.072	2.46 (0.73–8.32)	0.149
Genetics high risk	1.67 (0.65–4.29)	0.288	0.66 (1.19–2.24)	0.502
Mir-15a	0.26 (0.09–0.71)	0.008	0.28 (0.08–0.93)	0.038

Similar ROC curve analysis based on the cut-off value of 3.13 was performed for miR-16-1. Sixty-four patients with miR-16-1 < 3.13 were defined as low miR-16-1 expression group, while twenty-six patients with miR-16-1 ≥ 3.13 were defined as high miR-16-1 expression group. However, we didn't find any prognositic significance of miR-16-1 in newly diagnosed MM patients (data not shown).

### MiR-15a in combination with high β2-MG and high risk genetic abnormality can further predict high-risk subgroups in newly diagnosed MM patients

Based on the assumptions derived from the previous studies that miRNAs can be a valuable factor for improving ISS/FISH-based risk stratification, we next investigated if miR-15a in combination with other factors has the potential to predict the high-risk subgroups. Surprisingly, we found that miR-15a in combination with β2-MG or high risk genetic abnormality was a good predictor for dividing patients into subpopulations with different PFS and OS time (Table 4). Patients with low miR-15a expression (<2.35) and high β2-MG had worse PFS (15.0 months *vs*. 18.0 months *vs*. not reached, *P* = 0.012) and OS (15.0 months *vs*. 26.0 months vs. not reached, *P* = 0.001) than the patients either with high miR-15a expression and low β2-MG or other patients (Table 4, Figure 3C and 3D). A similar analysis for the PFS and OS prediction was also performed on the parameters of miR-15a and high risk genetic abnormality (Table 4, Figure 3E and 3F) and the data revealed that low miR-15a expression and high risk genetic abnormality are associated with the worst outcomes. Based on β2-MG ≥ 5.5 mg/dL being defined as ISS III stage, thus we thought miR-15a in combination with ISS III stage or high risk genetic abnormality could further select the high-risk subgroups in newly diagnosed MM patients. These high-risk subpopulations would have inferior clinical outcome than those with sole abnormality and thus require more positive therapies.

**Table 4:** Mir-15a combined with high β2-MG, high-risk genetic abnormality can further identify the high-risk subgroups in newly diagnosed MM patients

Subgroups	Median PFS (months)	*P* value	Median OS (months)	*P* value
**Mir-15a and β2-MG**		0.012		0.001
High mir-15a and low β2-MG (*n* = 26)	Not reached		Not reached	
Others (*n* = 52)	18.0		26.0	
Low mir-15a and high β2-MG (*n* = 8)	15.0		15.0	
**Mir-15a and genetics high risk**		*P* < 0.001		0.004
High mir-15a and no genetics high risk (*n* = 51)	29.0		Not reached	
Others (*n* = 22)	20.0		23.5	
Low mir-15a and genetics high risk (*n* = 5)	7.0		21.0	

### Bortezomib-based therapy can't improve the clinical outcome of myeloma patients with low miR-15a expression

**Figure 4:**
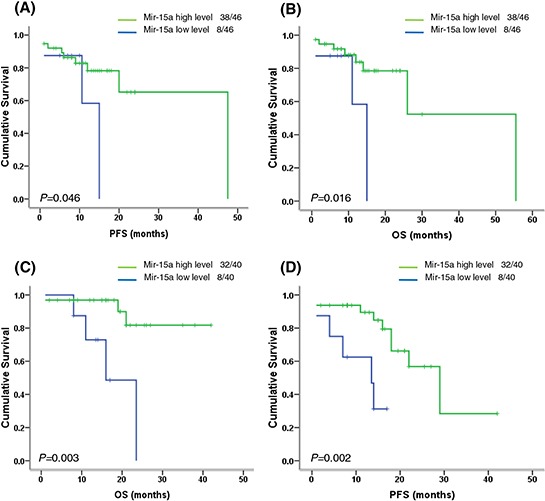
Survival analysis of miR-15a in newly diagnosed MM patients with thalidomide-based or bortezomib-based therapy **A–B.** In Arm A (thalidomide-based therapy), the patients with low miR-15a expression had inferior PFS (*P* = 0.046) and OS (*P* = 0.016). **C–D.** In Arm B (bortezomib-based therapy), the patients with low miR-15a expression also showed inferior PFS (*P* = 0.003) and OS (*P* = 0.002), suggesting that bortezomib-based therapy could not improve the poor survival of patients with low miR-15a expression.

Our data have showed the low expression of miR-15a was associated with disease progression and poor prognosis in MM patients. We further investigated whether miR-15a downregulation influenced the patients' response to different therapies. Interestingly, we found patients with low miR-15a expression had poor survival in patients received thalidomide (Arm A) and/or bortezomib (Arm B)-based chemotherapy (Table 5, Figure 4A–4D), suggesting that bortezomib-based treatment didn't significantly improve PFS and OS of patients with miR-15a low expression.

**Table 5:** Down-regulated mir-15a predicted poor survival in newly diagnosed MM patients receiving both thalidomide and bortezomib based therapy

Subgroups	Median PFS (months)	*P* value	Median OS (months)	*P* value
**Arm A**		0.046		0.016
Mir-15a low level (*n* = 8)	15.0		16.0	
Mir-15a high level (*n* = 38)	47.0		55.5	
**Arm B**		0.003		0.002
Mir-15a low level (*n* = 8)	13.5		16.0	
Mir-15a high level (*n* = 32)	29.0		Not reached	

## DISCUSSION

The importance of miRNAs in regulating critical genes associated with tumorigenesis, disease progression and drug resistance has been emphasized by many different published studies, and their roles as valuable diagnostic and prognostic biomarkers in MM has also been highlighted [13–15]. Moreover, these dysregulated miRNAs have been confirmed to be usually located at chromosome fragile sites that are involved in cancers [16]. The 13q14 deletion, one of the most frequent chromosomal aberration, occurs in 16–40% of multiple myeloma, more than or equal 50% of mantle cell lymphoma, chronic lymphocytic leukemia and other solid tumors [6]. Previous studies have reported that the expression of miR-15a/16-1 cluster, located at chromosome 13q14, is down-regulated in multiple pathological processes and it functions as a tumor suppressor gene in MM [9–12]. However, whether there is difference about their expression patterns and clinical roles in MM patients remains elusive. So we investigated the expression status of miR-15a/16-1 and assessed their diagnostic and prognostic values in myeloma patients.

Our results are consistent with previous published reports that miR-15a and 16-1 are universally down-regulated in newly diagnosed MM patients compared to HDs. Interestingly, although located at tightly linked site (13q14), miR-15a and 16-1 had different expression patterns. MiR-15a showed more variable expression levels than miR-16-1 in newly diagnosed patients. It was down-regulated in newly diagnosed and relapsed patients, however restored close to HDs level at the remission phase. Moreover, the patients with shorter PFS and OS had lower levels of miR-15a. However, the expression of miR-16-1 was also down-regulated in newly diagnosed and relapsed patients, but the level of miR-16-1 in the patients at remission phase did not restore to HDs levels. In addition, we also did not find any correlation among clinical characteristics and miR-16-1 expression. To assess the potential of miR-15a and miR-16-1 in distinguishing MM patients from HDs, we performed ROC analysis. Interestingly, we found that the AUC of miR-16-1 was 0.864, with 100% of sensitivity and 73% of specificity, while miR-15a had a AUC of 0.664. These results suggest that miR-15a is mainly associated with disease progression while miR-16-1 could be used as a good diagnostic marker in MM.

Several studies have also reported that the expression levels of miR-15a/16-1 are independent of the chromosome 13 status [17–19]. On the contrary, Roccaro *et al*. [20] found that patients with del(13q) had significantly decreased miR-15a/16-1 expression compared to patients without del(13q). But our data are in agreement with the previous conclusion that miR-15a/16-1 expression in MM patients is independent of the deletion of chromosome 13q14. The reasonable explanation is that most of the 13q14 deletions are heterozygous loss and they may have a limited impact on the expression levels of miR-15a/16-1. In addition, miR-15a/16-1 dysregulation resulted from chromosomal instability may be another reason.

Based on the correlation between miR-15a downregulation and disease progression and patient survival, we also compared the prognostic value of low miR-15a expression in newly diagnosed patients. Our data indicated that patients with low miR-15a expression (<2.35) had a significantly shortened PFS and OS than patients with high miR-15a expression (≥2.35). In multivariate analysis, miR-15a still remains an independent prognostic factor for short PFS and OS. Wu *et al*. [5] suggested that miRNAs can be built into molecular diagnostic strategies for improving the ISS/FISH-based risk stratification, our data also indicated that miR-15a in combination with high β2-MG or high risk genetic abnormality was a good prediction marker for classifying patients into high-risk subpopulations. These results further verify that miR-15a is a powerful prognostic marker in newly diagnosed MM patients.

It has been demonstrated that miR-15a/16-1 is down-regulated and functions as a tumor suppressor in MM. Roccaro *et al*. [20] identified that miR-15a/16-1 regulated the proliferation of MM cells *in vitro* and *in vivo* by inhibiting AKT serine/threonine protein kinase (AKT3), ribosomal-protein-S6, MAP-kinases, and NF-kB activator MAP3KIP3. Several other studies also revealed that miR-15a/16-1 targeted multiple genes that are related to cell cycle, apoptosis and angiogenesis, such as BCL2, MCL1, CCND1, WNT3A and VEGF [21]. However, whether there are different roles between miR-15a and 16-1 in the pathogenesis of MM remains unclear. In this study, our results indicated that miR-15a and 16-1 had different expression patterns and clinical values. Patients with lower miR-15a expression (<2.35) had inferior survival and were even resistant to bortezomib-based therapy, supporting that miR-15a plays more important roles in the disease progression and drug resistance compared to miR-16-1 in MM. Although the mechanism is unknown, this discrepancy can be explained by the following evidences: 1) our previous studies have indicated that bone marrow stromal cells decrease the sensitivity of myeloma cells to bortezomib treatment by more obviously downregulating the expression of miR-15a than miR-16-1, suggesting that miR-15a plays more important role in the drug resistance to bortezomib [11, 12]. 2) Roccaro *et al*. [20] investigated the clinical relevance of miR-15a/16-1 with ISS stage in MM, their results showed patients with ISS II and III had obviously lower levels of miR-15a but not miR-16-1, which provides the evidence for different roles of miR-15a/16-1 in MM. In future, different mechanisms of miR-15a/16-1 in the pathogenesis and drug resistance in MM need further investigation.

In summary, our results indicate that miR-15a and 16-1 are down-regulated in newly diagnosed myeloma patients but presented with different expression patterns. MiR-15a is associated with disease progression and poor prognosis while miR-16-1 may be a good diagnostic marker in MM. The results of our study also provide some important evidence for the potential clinical applications of miR-15a/16-1.

## MATERIALS AND METHODS

### Patients and samples

The study was approved by the ethics committee of the Institute of Hematology, Chinese Academy of Medical Sciences, and Peking Union Medical College, according to the guidelines of the 1996 Helsinki Declaration. Bone marrow samples were obtained from 90 newly diagnosed, 11 relapsed and 16 remission (≥VGPR, very good partial remission) MM patients. The normal bone marrows from 19 healthy donors (HDs) were collected as controls. Written informed consents from all patients and HDs were obtained. Diagnostic and response criteria for MM were referred to the recommendation by the International Myeloma Working Group (IMWG) [22].

Treatment regimens were performed as described in previous study [23], including thalidomide-based therapy (Arm A): TAD (thalidomide, adriamycin and dexamethasone) or TCD (thalidomide, cyclophosphamide and dexamethasone); and bortezomib-based therapy (Arm B): BCD (bortezomib, cyclophosphamide and dexamethasone) or PAD (bortezomib, adriamycin and dexamethasone). After at least four cycles of treatments with partial remission or better efficacy, patients underwent consolidation therapy with the patient's original regimen. Subsequently, patients were treated with thalidomide (100–150 mg/d) for one year to maintain response.

### Fluorescence *in situ* hybridization (FISH)

Mononuclear cells from different disease status of MM patients were separated by gradient density centrifugation. Plasma cells were then sorted using CD138-coated magnetic beads, which enabled plasma cell purity higher than 90%. Plasma cells were analyzed for the following chromosomal aberrations; del(13q14), del(17p), amp(1q21), IgH translocation, t(11;14), t(4;14), and t(14;16). FISH analysis was performed as reported previously [24]. At least 200 cells with well-delineated signals were evaluated. The cut-off level was set at 20% for numerical abnormalities and 10% for fusion or break-apart probes according to the recommendation by the European Myeloma Network (EMN) [25].

### Quantitative RT-PCR for miR-15a/16-1

Total RNA was extracted from purified CD138^+^ cells in a single step method using the mirVana™ miRNA isolation kit (Ambion, Austin, TX). The expression of miR-15a/16-1 was measured using the TaqMan microRNA quantitative PCR system (Applied Biosystems, Rotterdam, The Netherlands). The 10 ng of total RNA was reverse transcribed using the microRNA reverse transcription kit (Applied Biosystems) and a specific reverse transcription stem-loop primer according to the manufacturer's recommendations. All reactions were run in duplicate. Normalization was performed with RNU48. DC_t_ was calculated by subtracting the C_t_ value of RNU48 (Applied Biosystems) from the C_t_ value of miR-15a/16-1. The relative quantitative (RQ) values of miR-15a/16-1 were expressed as fold change of the miR-15a/16-1 relative to the expression in the MM cell line NCI-H929.

### Statistical analysis

The Log10+3 transformation of RQ value of miR-15a/16-1 was used in this analysis. Receiver operating characteristic (ROC) curves were used to determine the miR-15a/16-1 expression cut-off value. Categorical variables were compared using nonparametric tests and the Pearson's Chi-square test. Progression-free survival (PFS) was calculated from the date of diagnosis until disease progression or death, and overall survival (OS) was calculated from the date of diagnosis until death. Survival curves were plotted by the Kaplan-Meier method and differences between the curves were analyzed for statistical significance using the log-rank test. Multivariate analysis of variables associated with survival was conducted by cox proportional-hazard model for both PFS and OS. A statistically significant difference was considered at *P* ≤ 0.05. Statistical analysis was conducted using SPSS version 19.0 software.

## SUPPLEMENTARY TABLES


