# Supplementary material for: MicroRNA-15a/16-1 cluster located at chromosome 13q14 is down-regulated but displays different expression pattern and prognostic significance in multiple myeloma
Source: Oncotarget. 2015 Oct 12;6(35):38270–82. doi: 10.18632/oncotarget.5681 (PMC4741998; doi:10.18632/oncotarget.5681)
Supplement: Supplementary file 1 [file oncotarget-06-38270-s001.pdf]

SUPPLEMENTARY TABLES

Supplementary Table S1: The clinical characteristics of 90 newly diagnosed MM patients

Characteristics	Arm A<br>*N* = 49	Arm B<br>*N* = 41	*P*
Age (years), median (range)	58 (40–77)	58 (44–79)	0.524
Sex, F/M	19/30	16/25	0.981
Isotype, N (%)			
IgG	26/49 (53.1)	18/41 (43.9)	0.07
IgA	17/49 (34.7)	9/41 (21.9)	
IgD	1/49 (2.0)	5/41 (12.2)	
Light chains	5/49 (10.2)	9/41 (22.0)	
ISS stage, N (%)			0.789
I	5/46 (10.9)	6/40 (15.0)	
II	15/46 (32.6)	11/40 (27.5)	
III	26/46 (56.5)	23/40 (57.5)	
β2-microglobulin (mg/dL)	5.59 (2.3–39.1)	6.2 (1.4–23.9)	0.749
Durie-Salmon stage, N (%)			0.312
I - II	3/45 (6.7)	7/41 (17.1)	
III	42/45 (93.3)	34/41 (82.9)	
BM plasmacytosis (%), median (range)	34.0 (5.0–87.5)	25.8 (3.0–86.5)	0.566
Renal lesion, N (%)			0.119
None	38/46 (82.6)	28/41 (68.3)	
Present	8/46 (17.4)	13/41 (31.7)	
Cytogenetic abnormalities, N (%)			
Del(13q)	16/49 (32.7)	14/41 (34.1)	0.881
Del(17p)	3/47 (6.4)	7/41 (17.1)	0.115
IGH translocation	29/46 (63.0)	23/40 (57.5)	0.600
t(11;14)	7/41 (17.1)	7/39 (17.9)	0.918
t(4;14)	7/41 (17.1)	9/39 (23.1)	0.502
t(14;16)	1/41 (2.4)	4/38 (10.5)	0.140
Amp(1q21)	23/44 (52.3)	21/38 (55.3)	0.787
High-risk [(any t(4;14), t(14;16) or del(17p)]	9/43 (20.9)	13/39 (33.3)	0.206

F, female; M, male; ISS, International Staging System; Ig, immunoglobulin; BM, bone marrow; Arm A, thalidomide-based therapy; Arm B, bortezomib-based therapy.

Supplementary Table S2: The clinical characteristics and the remission status in 16 remission MM patients

	Age (years)	Isotype	D-S stage	ISS stage	Cytogenetic abnormalities	Treatment	Status of disease	miRNA-15a	miRNA-16-1
Case 1	63	IgA λ	III	II	Del(13q); t(4;14)	Arm B	CR	2.68	2.72
Case 2	42	IgG κ	III	I	Del(13q); amp(1q21); t(4;14)	Arm B	VGPR	3.83	2.47
Case 3	49	IgG κ	III	II	Amp(1q21); t(11;14)	Arm B	VGPR	3.43	3.2
Case 4	44	IgG κ	II	I	Negtive	Arm B	VGPR	4.5	3.6
Case 5	43	IgG κ	III	I	Negtive	Arm B	VGPR	4.65	3.99
Case 6	63	IgA λ	III	I	Negtive	Arm A	VGPR	3.53	1.87
Case7	45	IgA λ	III	II	Negtive	Arm B	CR	2.33	2.4
Case 8	61	IgAκ	III	III	Del(13q); t(4;14)	Arm B	CR	4.88	4.15
Case 9	51	IgG κ	III	II	t(4;14)	Arm B	VGPR	4.12	4.5
Case 10	53	IgD λ	III	III	t(11;14)	Arm B	CR	3.49	3.68
Case 11	59	κ	III	II	Negtive	Arm A	CR	3.95	3.78
Case 12	34	IgG κ	III	I	Del(13q); t(4;14)	Arm B	VGPR	3.78	3.13
Case 13	61	IgA λ	III	III	Del(13q); del(17p); IgH	Arm A	VGPR	4.29	2.64
Case 14	58	IgAκ	III	III	Negtive	Arm A	VGPR	3.52	2.6
Case 15	43	IgGλ	III	I	Amp(1q21)	Arm B	CR	2.68	3.17
Case 16	68	IgG κ	II	II	t(11;14)	Arm B	VGPR	3.48	3.31

D-S stage, Durie-Salmon stage; Arm A, thalidomide-based therapy; Arm B, bortezomib-based therapy; CR, complete remission; VGPR, very good partial remission
